# Role of Symmetry Breaking in Observing Strong Molecule–Cavity Coupling Using Dielectric Microspheres

**DOI:** 10.1021/acs.nanolett.2c02274

**Published:** 2022-08-03

**Authors:** Adarsh B. Vasista, Eduardo J. C. Dias, F. Javier García de Abajo, William L Barnes

**Affiliations:** ‡ Nanophotonic Systems Laboratory, Eidgenössische Technische Hochschule (ETH) Zürich, Zürich 8092, Switzerland; § Department of Physics and Astronomy, University of Exeter, Exeter EX44QL, United Kingdom; ⊥ ICFO-Institut de Ciencies Fotoniques, The Barcelona Institute of Science and Technology, Castelldefels, Barcelona 08860, Spain; || ICREA-Institució Catalana de Recerca i Estudis Avançats, Passeig Lluís Companys 23, Barcelona 08010, Spain

**Keywords:** symmetry breaking, strong coupling, Mie scattering, dielectric cavities, J-aggregates

## Abstract

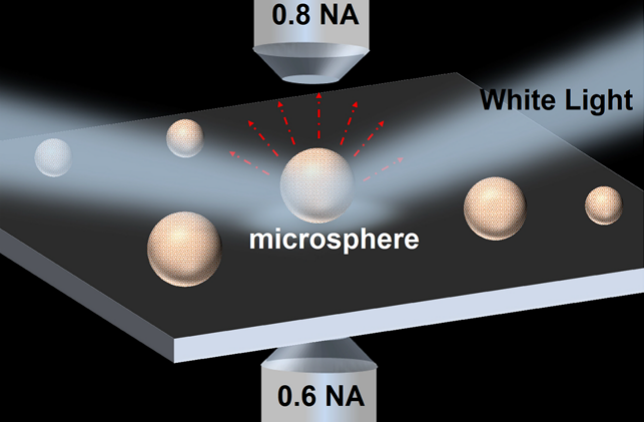

The emergence of dielectric open optical cavities has opened a new research avenue in nanophotonics. In particular, dielectric microspheres support a rich set of cavity modes with varying spectral characteristics, making them an ideal platform to study molecule–cavity interactions. The symmetry of the structure plays a critical role in the outcoupling of these modes and, hence, the perceived molecule–cavity coupling strength. Here, we experimentally and theoretically study molecule–cavity coupling mediated by the Mie scattering modes of a dielectric microsphere placed on a glass substrate and excited with far-field illumination, from which we collect scattering signatures both in the air and glass sides. Glass-side collection reveals clear signatures of strong molecule–cavity coupling (coupling strength 2*g* = 74 meV), in contrast to the air-side scattering signal. Rigorous electromagnetic modeling allows us to understand molecule–cavity coupling and unravel the role played by the spatial mode profile in the observed coupling strength.

The study of systems with broken symmetry is vital in the discovery of many exotic optical phenomena.^1^ Broken symmetry can drive the creation of new energy eigenstates in materials,^2,3^ the generation of chiral responses,^4−6^ the boosting of Fano resonances,^7^ the emergence of the plasmonic spin-Hall effect,^8^ and the existence of topological superconductors.^9^ The modal structure of certain cavities, like dielectric microspheres, depends critically on their symmetry and local environment. These so-called morphology-dependent resonances have been utilized to detect single molecules,^10^ determine the size of nanoparticles,^11^ and even strongly couple single atoms.^12^

It is well-known that the way in which molecules absorb, emit, and transfer energy can be drastically modified by coupling them to optical cavities. In particular, strong molecule–cavity coupling leads to a radical modification of the molecular energy landscape and may result in the emergence of new properties, such as threshold-less lasing,^13,14^ long-range energy transfer,^15^ and tilted ground-state reactivity,^16^ among others. In this regard, probing molecule–cavity coupling with morphology-dependent resonances is extremely relevant.

The cavities commonly employed to achieve strong coupling can be classified into two types: open cavities, where molecules can be adsorbed and desorbed easily, and closed cavities, where there is little room for dynamic molecular movement. Both open and closed cavities have their own advantages and limitations. In the context of strong coupling applications, open cavities provide the technological advantage of accessibility to the molecular medium. Various optical–cavity architectures have been studied in the past to strongly couple molecules, such as metallic nanostructures,^17^ dielectric nanoparticles,^18^ microtoroids,^12^ and microspheres.^19^ Because of the negligible inelastic losses and the existence of multiple cavity modes, dielectric structures such as microspheres are often considered advantageous over their metallic counterparts.

Dielectric microspheres support a multitude of electromagnetic resonances called Mie modes. They are characterized by the radial (*n*), orbital (*l*), and azimuthal (*m*) quantum numbers, as well as the polarization symmetry (TE or TM). Here, *n* and *l* are positive integers and *m* is an integer that takes values from −*l* to *l*. The spatial electromagnetic field profile of the Mie resonances inside the microsphere is expressed in terms of spherical Bessel functions *j*_*l*_ multiplied by spherical harmonics. For high orbital quantum numbers *l*, the Bessel functions have a maximum amplitude near the periphery of the microsphere. In addition, a Mie mode with orbital quantum number *l* has a 2*l* + 1 degeneracy in the azimuthal quantum number *m*. These delocalized, high-*l* modes are termed whispering gallery modes (WGMs) because of their resemblance to the well-known acoustic modes of a similar nature.

Strong molecule cavity coupling has been reported with WGMs in the past,^19,20^ thus raising the question of whether can we generalize the concept and strongly couple molecules to all the Mie scattering modes of a microsphere. The answer to this question has wide implications, not only for the design of open cavities for polariton-chemistry applications but also in understanding the physics of strong coupling in multimode cavities. Additionally, as the spectral characteristics of the Mie modes depend critically on the symmetry of the structure, this question poses a unique opportunity to study the effect of structural symmetry on molecule–cavity coupling in open systems. Symmetry plays a key role in defining the molecule–cavity coupling strength, and its detailed study provides a deeper understanding of the physics of strong coupling.^21^ Additionally, in multimodal cavities, strong coupling can introduce decoupling of modes,^22^ emphasizing the importance of studying in detail the physics of strong coupling in such cavities.

The coupling efficiency to WGMs using plane-wave excitation is low, as the efficiency decreases for large values of the orbital quantum number *l*, albeit remaining nonzero.^23^ Hence, one can excite WGMs with far-field excitation, but with small efficiency. Interestingly, the coupling efficiency can be enhanced by matching the effective mode velocity with the phase velocity of an evanescent source. This makes evanescent-wave-based sources the preferred excitation mechanism to generate WGMs, although there are also reports on the excitation of WGMs with far-field sources.^24,25^

With this motivation in mind, we coupled four layers of S2275 dye molecules to an individual polystyrene microsphere of radius ∼1.5 μm using a layer-by-layer deposition method.^26^ The microsphere was then placed on a glass substrate and probed using dark-field scattering spectroscopy. Keeping in mind that the presence of a substrate strongly alters the spectroscopic signatures of the outcoupled modes, we collected the scattered light both in the air and in the glass sides. Interestingly, we found significant differences in the spectral characteristics of the outcoupled modes and their molecule–cavity coupling strengths for air- and glass-side collection. The Mie scattering modes collected in the glass side show strong molecule–cavity coupling, while the ones in the air side do not. These experimental findings are supported by numerical simulations based on a semianalytical, rigorous solution of Maxwell’s equations for a spherical scatterer on a planar substrate.^27,28^

A schematic representation of the system under study is shown in Figure 1a. Individual dye-coated microspheres are probed in the dark-field configuration by exciting them with a white light source under oblique incidence. The scattered light from the microspheres is separately collected both in the air side and in the glass side, and then analyzed as a function of wavelength (see Section S1 for details of the experimental setup and the sample preparation). The incident light polarization is parallel to the substrate plane. To understand the spectral response of bare dye molecules, we measured the absorption spectrum of 4 layers of S2275 dye molecules coated on a planar glass substrate, as shown in Figure 1b. The absorption shows a prominent peak around 650 nm, which corresponds to the absorption of J-aggregated dye, whereas monomer peaks show up as weaker features at lower wavelengths (see Section S1 for further details). Because of the weaker oscillator strength and larger line widths, the monomer peaks do not contribute significantly to the molecule–cavity coupling centered around the 650 nm molecular resonance. However, the J-aggregate absorption plays a critical role, with the absorption line width determined to be 67 ± 1 meV.

**Figure 1:**
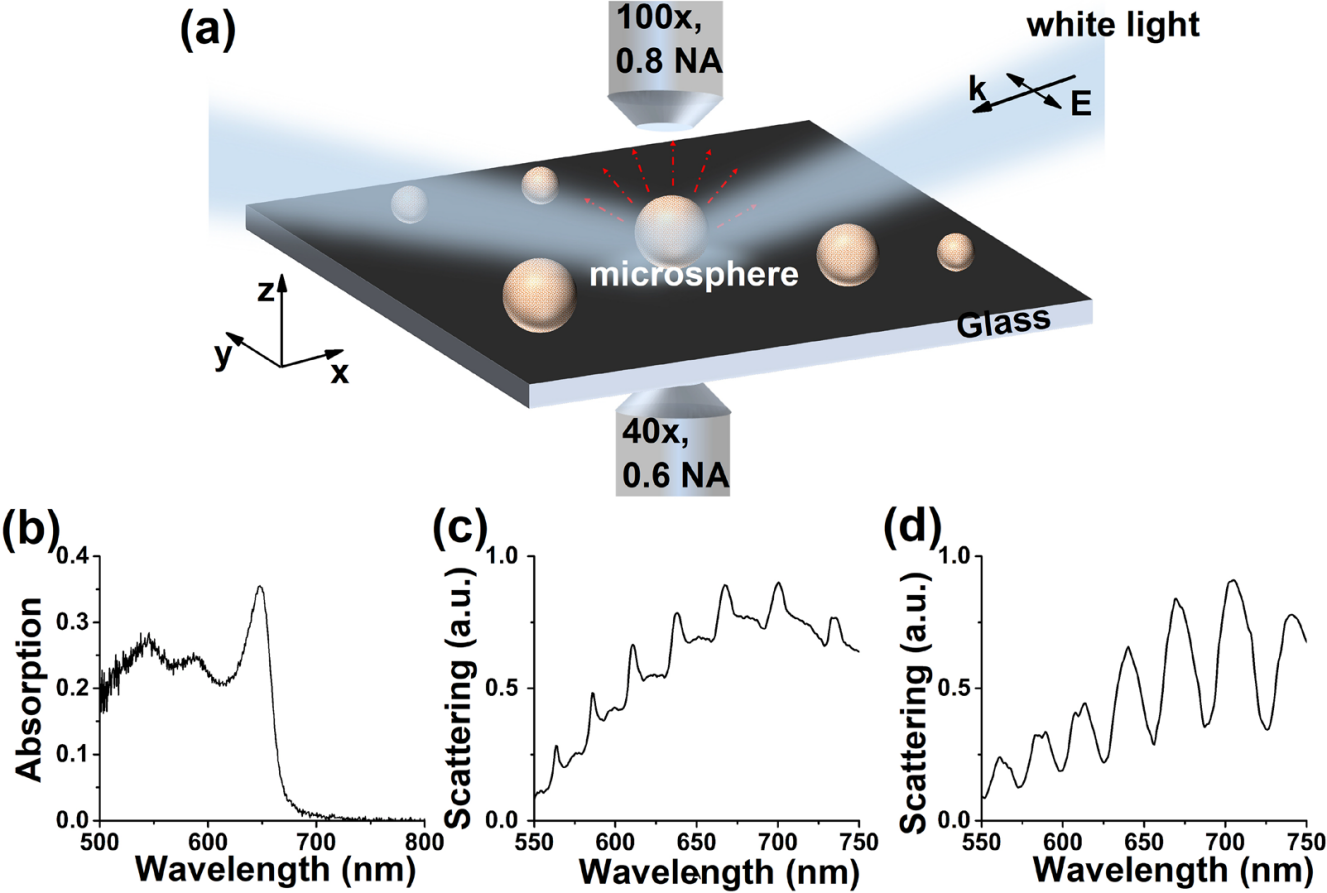
(a) Schematics of the experimental configuration used in this work. An individual dye-coated microsphere is excited using white light incident at an oblique angle. The resulting light scattering is collected and spectrally analyzed in both in the air and the glass sides. (b) Experimentally measured absorption spectra of four layers of S2275 dye molecules deposited on a glass substrate. (c, d) Measured scattering spectra of an individual microsphere of radius ∼1.5 μm as acquired from either (c) the glass side or (d) the air side.

When a multimodal scatterer is placed on a substrate, the mismatch of refractive indices introduces a selective outcoupling of the modes. This phenomenon has been studied extensively in plasmonic scatterers such as nanowires.^29,30^ Microspheres also support a multitude of Mie modes so that the outcoupling of the modes depends on the local environment of the microsphere. For a microsphere placed on a planar substrate, the broken symmetry results in different spectral properties of the signal emanating from Mie modes through the air and glass sides. Figure 1c shows the scattering spectrum of an individual microsphere collected in the glass side, where we observe spectrally sharper resonances with a line width of 25 ± 1 meV. In contrast, the scattering spectrum of an individual microsphere collected in the air side, plotted in Figure 1d, exhibits a broader measured line width (80 ± 2 meV). For completeness, we also study WGMs in dye-coated microspheres in the context of molecule–cavity coupling (see more details in Section S2).

Figure 2a, b shows the dispersion of Mie scattering modes collected in the air side in the absence and presence of a coating dye layer. We performed experiments on multiple microspheres, each microsphere having a slightly different radius (all around ∼1.5 μm) and arrange the measured spectra in the order of ascending radii to generate the dispersion plot shown in Figures 2a, b. The size of the microspheres was estimated by fitting a part of the experimental spectrum with the corresponding numerically simulated one (see ref (19) for a detailed discussion on this procedure). Figure 2a shows the dispersion plot of scattering modes collected in the air side for bare microspheres, while Figure 2b shows for comparison the dispersion plot for microspheres coated with four layers of S2275 dye molecules. We observe that the scattering mode resonance is perturbed by the molecular resonance, particularly close to the 650 nm spectral region, where the dye is highly absorbent. However, we do not find any trace of strong molecule–cavity coupling (i.e., splitting of the scattering mode and an anticrossing profile).

**Figure 2:**
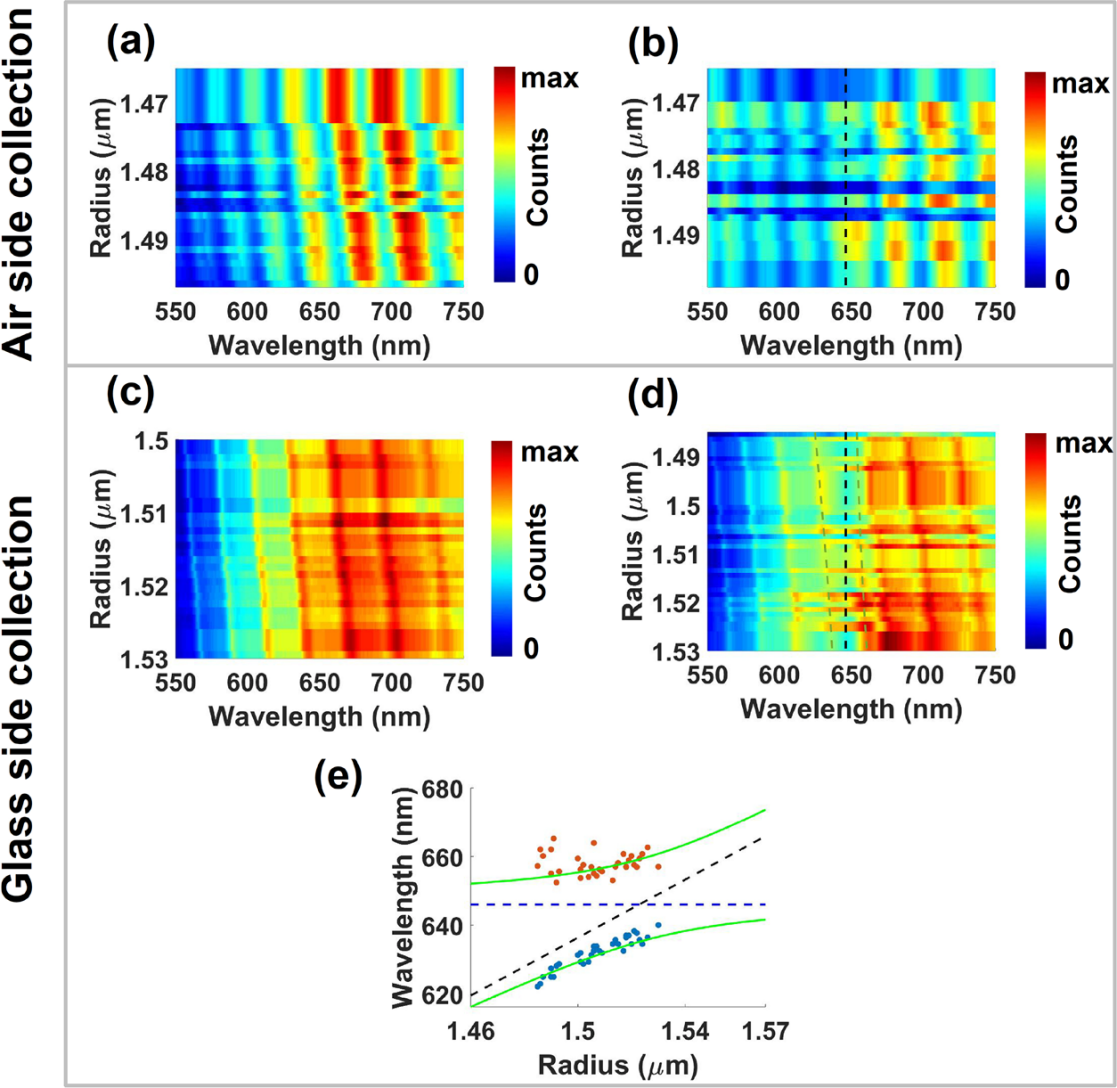
Experimentally measured dispersion of scattering modes collected in (a, b) the air side and (c, d) the glass side from (a, c) a bare individual microsphere and (b, d) an individual microsphere coated with four layers of S2275 dye. Black-dotted lines in b and d indicate the absorption maximum of the J-aggregated S2275 dye molecules. The dashed green lines represent the polariton energies calculated using the coupled oscillator matrix. (e) Dispersion plot of the scattering mode resonant with the S2275 dye absorption. Experimental values for the spectral positions of the lower and upper polariton branches are extracted from panel d and represented as dots. The green curves correspond to a fit using a coupled oscillator model. The black-dashed line represents the unperturbed scattering mode and the dashed-blue line is the absorption maximum wavelength of the J-aggregated S2275 dye molecules.

In contrast, mode leakage through the glass substrate reveals a different story, as shown in Figure 2c–e. This superstrate/substrate dependence is unique to the microspheres, as nano/microcavities previously used to couple molecules show only marginal spectral modifications depending on the excitation and collection configuration. Figure 2c shows the dispersion of scattering modes of a bare microsphere collected in the glass side. Comparing Figure 2a, c, we observe a clear difference of the spectral line widths between the signal resulting from outcoupling of the microsphere modes through the air and glass sides. Interestingly, when the microsphere is coated with four layers of S2275 molecules, we find a splitting of the cavity resonance due to hybridization with the resonant feature of S2275, as shown in Figure 2d. To quantify the coupling strength *g*, we fit the experimental data with a coupled oscillator model given by the secular matrix of the system
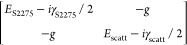
1where *E*_S2275_ = 1.919 eV is the photon energy for maximum absorption by the J-aggregated S2275 dye molecules, γ_S2275_ = 67 meV is the line width of the molecular absorption, *E*_scatt_ is the energy of the scattering mode of the microsphere, and γ_scatt_ = 25 meV is the line width of the scattering mode (which we assume to be approximately constant over the small range of microsphere radii measured). The eigenvalues of eq 1 represent the polariton energies, shown as green lines in Figure 2e. The coupling strength is found to be 2*g* = 74 ± 4 meV. The value of 2*g* was greater than the mean value of the molecular absorption line width (67 meV) and the cavity line width (25 meV), showing that the system operates in the strong coupling regime. Figure 2 sheds light on the interesting fact that, even though the conditions of excitation of the microsphere modes are not changed, the perceived molecule–cavity coupling strength depends on the collection configuration. The figure of merit (FOM) for strong coupling in cavities is defined by *Q*/*√V*, where *Q* is the quality factor of the cavity and *V* is the mode volume. For Mie modes, the spectral line widths of the Mie resonances collected on the glass side are relatively narrower than the ones collected on the air side, thus playing a critical role in the observed molecule–cavity coupling strengths in either side.

To better understand this difference in the apparent molecule–cavity coupling, we perform numerical calculations by rigorously solving Maxwell’s equations for a sphere placed on a planar substrate. The microsphere is excited using a plane wave incident at an angle of 20° with respect to the substrate. The incident plane wave field is then rigorously decomposed into a convergent superposition of spherical waves, whose scattering by the sphere is described analytically using Mie theory. The scattered far-field intensity is then integrated over a solid angle corresponding to the numerical aperture used in the experiment on either side of the sample (see Figure S1 for details), thus yielding a scattering efficiency *Q*_scatt_ akin to the number of measured photon counts. A detailed discussion on the simulation strategy, as well as details on the modeling of the optical response of the S2275 dye, can be found in the Section S3.

Figure 3 shows the numerically calculated spectrum of the scattering efficiency by a microsphere placed on a glass substrate upon excitation with white light under oblique incidence for (a, b) air-side and (c, d) glass-side collection, both of them in the (a, c) absence or (b, d) presence of a coating molecular layer. For the sake of clarity, we plot a larger range of microsphere radii than in the experimental data of Figure 2. For air-side collection (Figure 3a, b), the presence of the molecular coating results in a suppression of scattering at wavelengths close to the dye absorption peak (marked with a white-dashed line), but there is no clear evidence of strong molecule–cavity coupling, in agreement with the experimental observation discussed above (cf. Figures 2b and 3b). In contrast, for the glass-side collection (Figures 3c, d), we observe a clear splitting of the scattering mode in resonance with the molecular absorption peak, as indicated by the green-dashed lines in panel Figure 3d. To quantify this splitting, we fit the numerical data with a coupled oscillator model given by the secular matrix
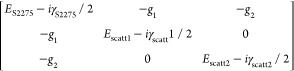
2where *E*_scatt1_ and *E*_scatt2_ are energies of Mie scattering modes that overlap with the molecular resonance (shown as white lines in Figure 3d), whereas *g*_1_ and *g*_2_ are their coupling strengths to the molecular resonance. The calculated coupling strength of the split mode is found to be 2*g*_2_ = 80 ± 2 meV, which is a reasonable match to the experimental value.

**Figure 3:**
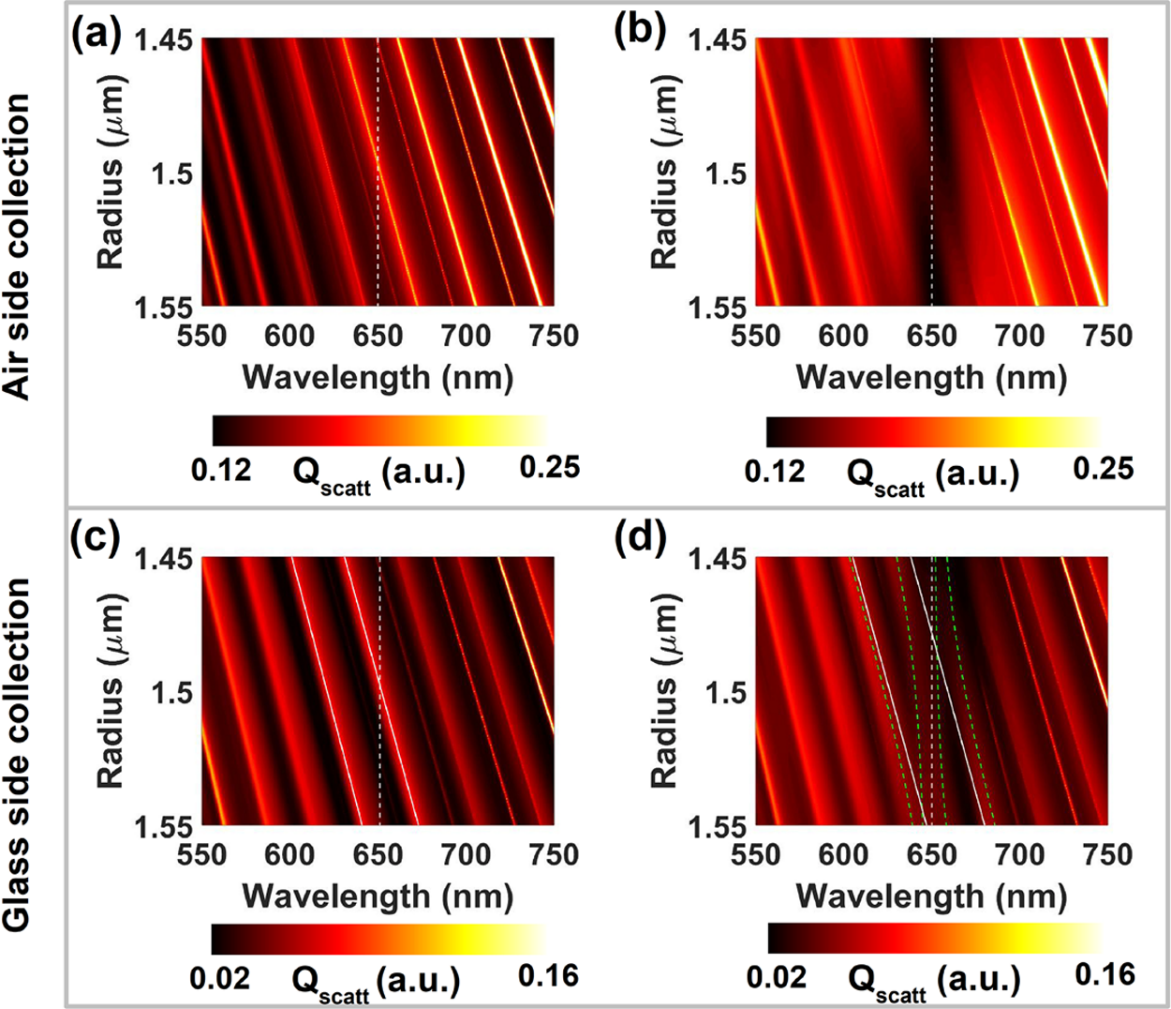
Calculated dispersion of the air-side scattering efficiency *Q*_scatt_ of (a) bare microsphere and (b) S2275-dye-coated microsphere deposited on a planar glass substrate. (c, d) Same as a and b, respectively, but for glass-side scattering. The dashed-white lines represent the wavelength for which the molecular absorption is maximum. In panel d, a coupled-oscillator model fit is superimposed as dashed-green lines to quantify the molecule–cavity coupling strength. Solid-white lines in panels c and d represent the bare cavity modes of the microsphere. The light-incidence angle is 20° with respect to the substrate in all cases.

It is important to note that the numerically calculated scattering spectra contain spectrally sharp resonance peaks, particularly for air-side collection, which are absent in the measured spectra (cf. Figures 3a and 2a). We suggest that in the experiment the sharp peaks are damped due to morphological imperfections in the microspheres, as well as by accounted losses and the finite spectral resolution of the light detector, which results in peak broadening. We try to mimic these effects by introducing a Gaussian blur to the numerically calculated spectra by convolving each wavelength with a Gaussian centered around it with varying full-width at half-maximum (fwhm). This procedure broadens the sharp peaks observed in Figure 3 and yields a better agreement with the experimental results of Figure 2, as shown in Section S3.

Figure 3b, d, along with Figure 2b, d, show that there is a clear difference between the molecule–cavity coupling strength associated with the Mie scattering modes in the air and glass sides. We attribute this difference to a strong symmetry breaking between the upper and lower hemispheres of the microsphere due to the presence of the substrate, which perturbs its local environment (i.e., the embedding media around the microsphere is not uniform), resulting in a selective outcoupling of the cavity modes.

For comparison, we also calculate the dispersion of scattering spectra of a microsphere in the absence of a substrate, as shown in Figure S9. The coated dye introduces a strong perturbation in the scattering modes, but due to smudging of the spectral information on the scattering modes, it is difficult to calculate molecule–cavity coupling strengths and we artificially reduced the line width of the molecular resonance to draw conclusions on molecule–cavity coupling. A discussion on this topic is given in Section S3. In contrast, when he microsphere is placed on a glass substrate, the broken structural symmetry plays a critical role in the outcoupling of modes, so that we can easily quantify the molecule–cavity coupling strength (cf. Figure 4).

**Figure 4:**
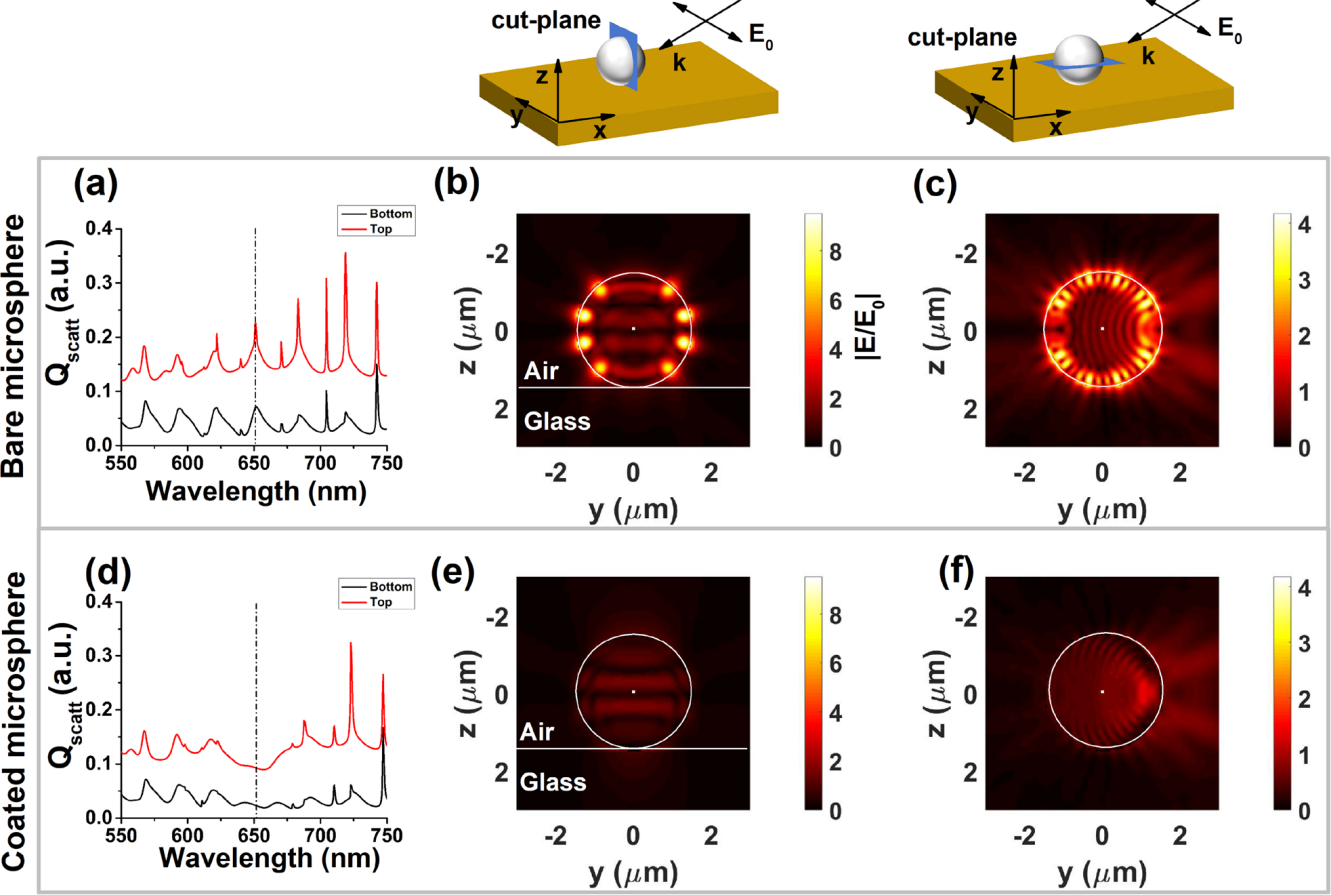
(a) Numerically calculated scattering spectra of a bare microsphere of radius 1.5 μm on a glass substrate for air-side (red-solid curve) and glass-side (black-solid curve) collection under illumination by plane-wave propagating as indicated in the upper schemes. The black-dashed line indicates the excitation wavelength of 651 nm used in the calculations of b, c, e, and f. (b, c) Electric-field enhancement profiles calculated along the cross-sectional planes indicated in the upper sketches. (d–f) Same as a–c, respectively, but for the microsphere coated with four layers of S2275 dye.

To obtain a better understanding of the effect of the dye coating on the supported microsphere, particularly in the ∼650 nm region where Figures 2 and 3 reveal a strong coating-induced effect, we use the same theoretical approach as above (see Section S4) to calculate the scattered near-field enhancement profile for a 1.5 μm radius microsphere placed on a glass substrate under plane wave illumination (see schemes in Figure 4 for geometrical details). Figure 4a, d shows the scattering efficiency *Q*_scatt_ for both air-side and glass-side collection conditions from individual bare and coated microspheres, respectively, clearly revealing the presence of a sharp resonance at 651 nm (indicated by a black-dashed line), which completely disappears in the presence of the molecular coating. Panels b and c show the near-field enhancement for two different cross-sectional cuts of the sphere (indicated in the upper schematics) in the absence of any coating, which are characterized by a strong field intensity in the vicinity of the sphere surface. Panels e and f show the respective counterparts of panels b and c, but in the presence of the dye coating. From the comparison of the two cases, we conclude that the dye (which is highly absorbent at the chosen wavelength) dramatically changes the electromagnetic boundary conditions at the surface of the sphere and suppresses the field intensity at the sphere surface, producing instead a concentration of the field near the sphere center. For comparison, we show in Figure S8 similar plots to those shown in Figure 4, but calculated for a wavelength away from the dye absorption resonance, showing that the effect of the dye in the electric near-field is very mild under these conditions.

To summarize, we experimentally and numerically studied the coupling of molecules with the Mie scattering modes of individual microsphere cavities placed on a glass substrate. We probed the effect of broken symmetry on the spectral characteristics of the microsphere and showed that modes outcoupling into the air side exhibit a different molecule–cavity coupling strength compared to those emitting into the glass side. We also performed rigorous numerical calculations that allow us to model the effect of broken symmetry on the line width of the Mie scattering modes and the deduced molecule–cavity coupling strengths. It is important to note that a seemingly simple microsphere placed on a glass substrate is in fact a complex system to model and to understand. A large microsphere supports multiple spectrally sharp Mie scattering modes, and accurately modeling the effect of the glass substrate on them is challenging, as simple morphological inconsistencies (such as any deviation from a perfect sphere) can lead to significant changes in the mode profiles. However, our model supports the experimental observations to a remarkable extent and provides substantial insight into the effect of broken structural symmetry on spatial and spectral signatures of the particle Mie modes. These results emphasize the unique, rich physics displayed by the rather simple structure of a dielectric microsphere. Our results also provide detailed understanding of molecular coupling in open cavities, which, beyond its fundamental interest, could trigger applications in optical sensing.

## Supporting Information Available

Experimental setup, additional details on numerical simulation, and a discussion on strong coupling using WGMs (PDF)
