# Supplementary material for: Role of Symmetry Breaking in Observing Strong Molecule–Cavity Coupling Using Dielectric Microspheres
Source: Nano Lett. 2022 Aug 3;22(16):6737–43. doi: 10.1021/acs.nanolett.2c02274 (PMC9413215; doi:10.1021/acs.nanolett.2c02274)
Supplement: Supplementary file 1 — nl2c02274_si_001.pdf [file nl2c02274_si_001.pdf]

Role of Symmetry Breaking in observing Strong Molecule-Cavity Coupling using Dielectric Microspheres

Adarsh B Vasista[1,2,*], Eduardo J. C. Dias[3], F. Javier García de Abajo[3,4], and William L Barnes[1,*]

[1]Department of Physics and Astronomy, University of Exeter, Exeter EX44QL, United Kingdom
[2] Nanophotonic Systems Laboratory, ETH Zurich, 8092 Zurich, Switzerland
[3]ICFO-Institut de Ciencies Fotoniques, The Barcelona Institute of Science and Technology, 08860 Castelldefels, Barcelona, Spain
[4]ICREA-Institució Catalana de Recerca i Estudis Avançats, Passeig Lluís Companys 23, 08010 Barcelona, Spain
[*] E-mail: avasista@ethz.ch; w.l.barnes@exeter.ac.uk

Contents

S1 Methods and experimental setup **2**
S1.1 Scattering-mode characterization through Fourier-plane imaging . . . 4

S2 Whispering gallery modes and strong coupling **5**

S3 Microsphere on a planar glass substrate: Numerical simulations **6**
S3.1 Peak broadening . . . 6
S3.2 Electric-field profiles . . . 9
S3.3 Molecule-cavity coupling in unsupported microspheres . . . 9

References **10**

S1 Methods and experimental setup

For the layer-by-layer fabrication, we used a cationic poly(diallyldimethylammonium chloride) (PDAC) solution as the polyelectrolyte binder for anionic S2275 J-aggregate solution. A typical deposition step consisted of mixing 20 μl PDAC solution (20% by weight in water - diluted 1:1000) with 1 ml of PS microsphere colloidal solution (15% by weight in water - 1:50) and allowing it to settle for 20 minutes. The solution was then washed 3 times in water to remove excess polyelectrolyte solution. This step formed a positively charged layer of PDAC on the microspheres. Then, a 100 μl S2275 solution (0.01 M) was added to the microsphere/PDAC colloid and kept for 20 minutes. The solution was then washed 3 times in water to remove excess S2275 molecules. This procedure was repeated to deposit multiple layers of J-aggregate S2275 on the surface of the microspheres. In this work, we have deposited 4 layers of molecules as a compromise between observing a significant Rabi splitting of the modes and, at the same time, not affecting the signal-to-noise ratio due to suppression of the scattering intensity. Finally, the S2275 layer was protected by depositing a layer of PDAC molecules. To increase the adhesion of the dye molecules, the polystyrene microspheres were first coated with a layer of anionic polystyrene sulfonate (PSS) using the layer-by-layer technique discussed above.

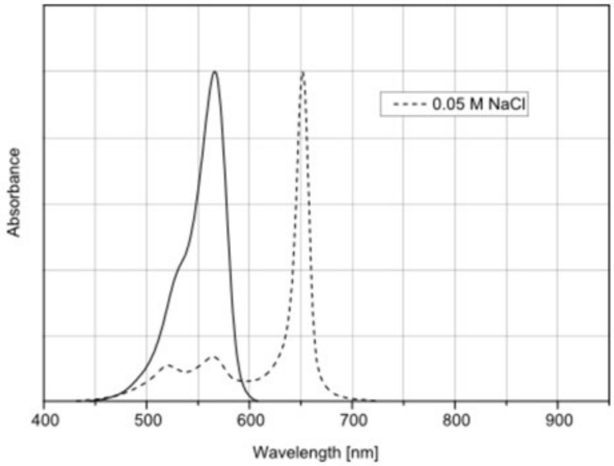

Figure S1: Absorption spectrum of S275 dye. Reproduced from FEW chemicals' data sheet on S2275 molecular absorption.

Monomer S2275 dye shows a relatively broad absorption at 566 nm with a shoulder at 530 nm. When the S2275 dye molecules are dissolved in 0.05M aq. solution of NaCl, the altered pH allows the molecules to aggregate, the absorption peak shifts to 650 nm, and the linewidth reduces, as shown in the manufacturer's data sheet reproduced as Figure S1.

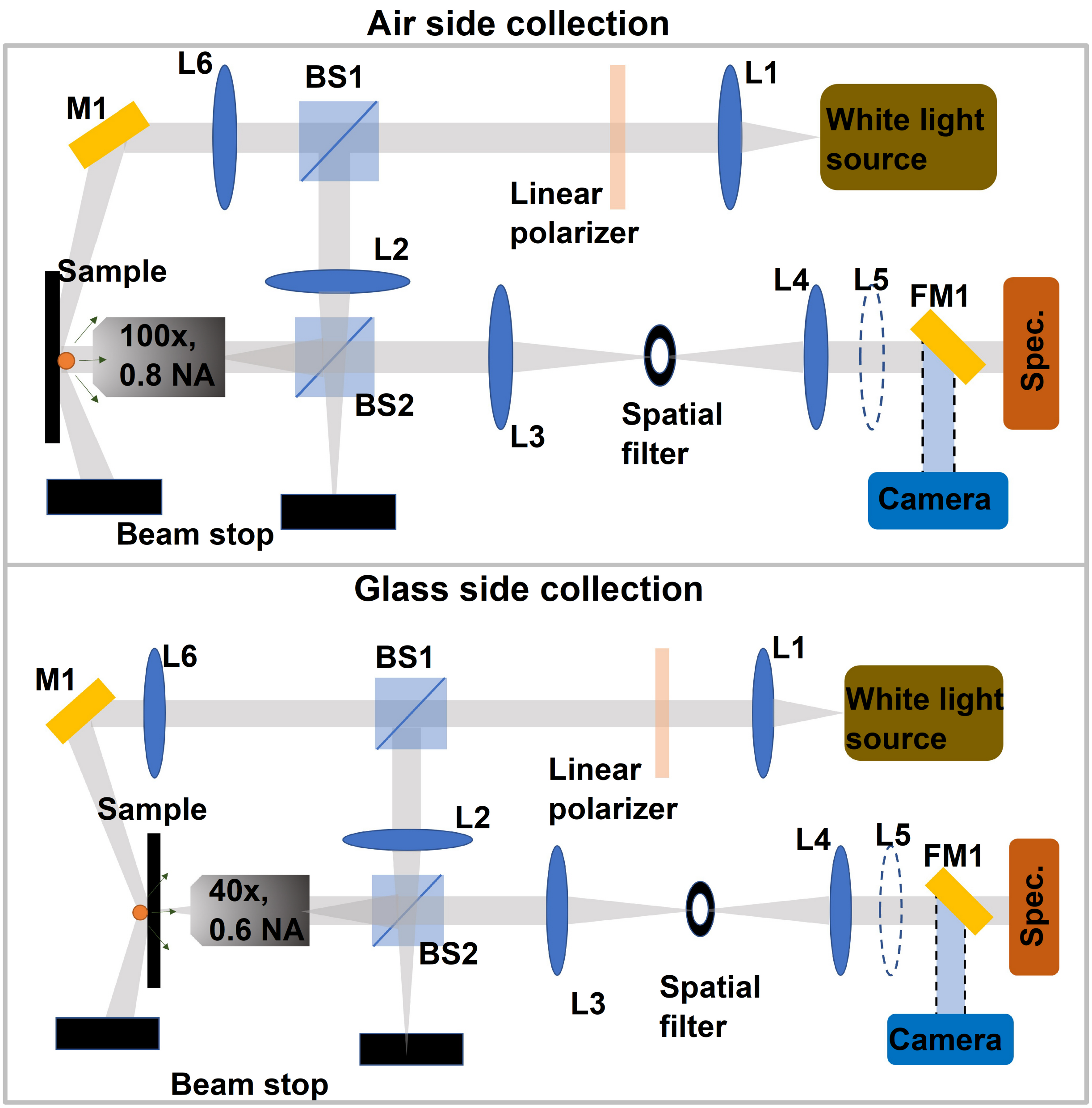

Figure S2: Scheme of the experimental setup.

Figure S2 shows a scheme of the experimental setup used to probe dark-field scattering from an individual microsphere placed on a glass substrate. The microsphere is excited using white light at an oblique angle. The scattered light is collected either in the air side through a 0.8 NA, 100x objective lens or in the glass side using a 0.6 NA, 40x objective lens (with a correction collar to compensate for the glass substrate thickness). The difference in the objectives in the glass and air sides is due to availability of these components, given the need of an objective lens with a built-in correction collar in the glass side to compensate for the spherical aberrations introduced by the substrate. Lenses L3 and L4 form a telescope configuration, while lens L5 projects the image plane onto the spectrometer or to the camera, depending on the position of the mirror FM1. An adjustable pinhole is placed at the conjugate image plane to spatially filter the scattering signal from an individual microsphere. Lens L1 is a collimator and Lens L6 focuses the light onto the sample plane. In both the cases, we have BS1 and BS2 are utilized for illuminating the sample through the objective lens for visualizing the microspheres.

S1.1 Scattering-mode characterization through Fourier-plane imaging

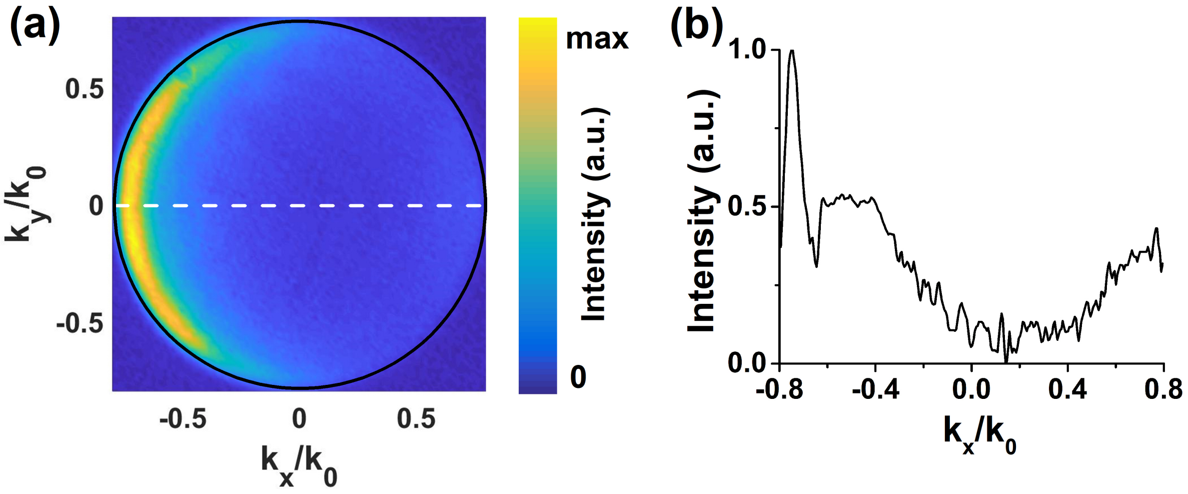

Figure S3: (a) Fourier-plane image of the scattered light in the air side showing directional scattering. (b) Line profile of the scattered light as a function of k_x/k_0 for k_y/k_0=0 (i.e., along the white-dotted line in (a)).

Figure S3(a) shows the Fourier-plane (FP) image of the scattered light in the air side. We observe that the microsphere scatters light directionally. This is an expected result because Mie scattering is directional and the bulk of the scattered light propagates along the direction of the incident light. As the illumination configuration involves oblique incidence, we see that most of the scattering emerges at high values of k_x/k_0. We quantify the angle of scattering by plotting a line profile of the FP image at $k_y = 0$, as shown in Figure S3(b). The scattering maximum happens at $k_x/k_0 = -0.75$, which corresponds to an angle of $-48.5°$.

S2 Whispering gallery modes and strong coupling

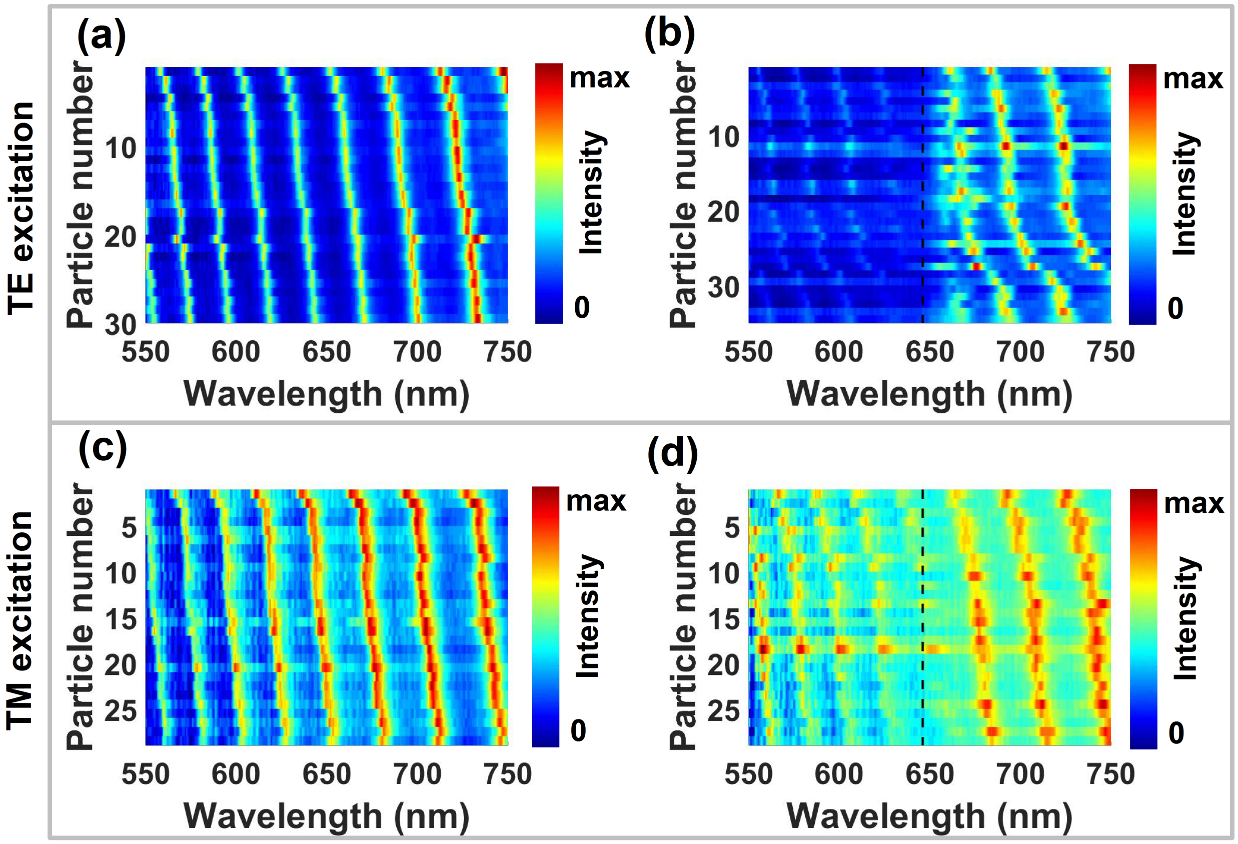

Figure S4: (a,b) Dispersion of whispering gallery modes of (a) a bare microsphere and (b) a microsphere coated with four layers of S2275 dye molecules for TE-polarized excitation. (c,d) Same as (a,b), respectively, but for TM-polarized excitation. The dashed-black line represents the molecular absorption maximum. The radius of the microsphere is $\sim 1.5\ \mu m$.

Figure S4 shows the dispersion of the whispering gallery modes of an individual microsphere placed on a glass substrate. To excite whispering gallery modes in the sphere, we utilize total internal reflection in a dark-field configuration, as explained in Refs. 1 and 2. For TE-polarized excitation, when four layers of S2275 dye molecules are deposited on the microsphere, the mode that is on resonance with the molecular absorption splits, as shown in Figures S4(a) and (b). However, for TM-polarized excitation, Figures S4(c) and (d) show a minimal perturbation when the molecular layer is deposited. This indicates a polarization mismatch between the sphere WGM and the dipoles associated with the molecular resonance, as the latter have zero radial dipole moment. It is important to note that spectral signatures of WGMs do not depend on the collection configuration unlike the Mie scattering modes.

S3 Microsphere on a planar glass substrate: Numerical simulations

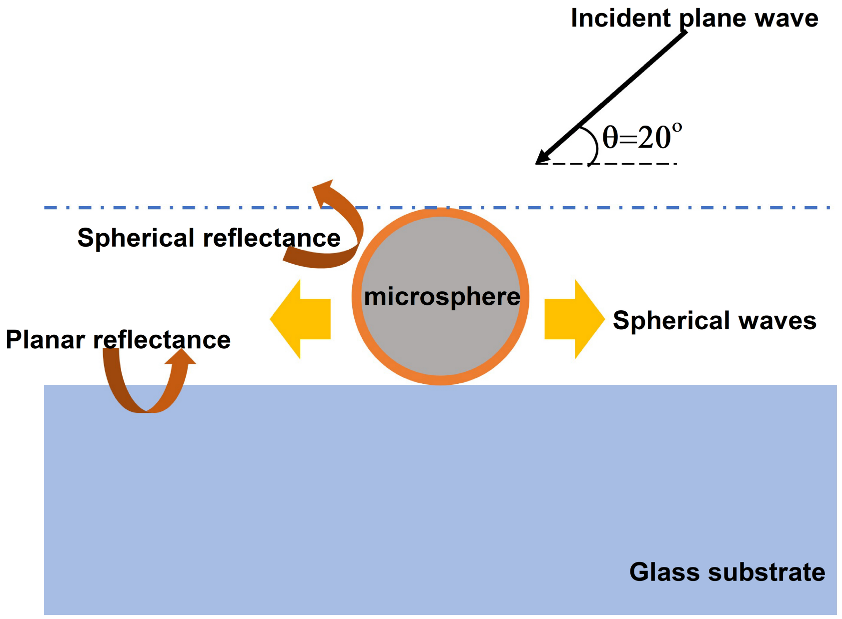

Figure S5: Schematic representing of the simulation strategy.

Calculating light scattering from a micrometer-sized spherical structure on a planar interface is a challenging task using commercial solvers such as COMSOL due to meshing and convergence issues. Instead, we rigorously solve Maxwell's equations by expanding the scattered field in terms of spherical waves in the sphere region and plane waves above and below there sphere. In essence, we adopt an analytical procedure to transform field expansions between both types of waves and further introduce scattering by the sphere through Mie theory for spherical waves and reflection by the surface through the Fresnel coefficients for plane waves. We use the procedure described in Refs. 3 and 4, which is based on the noted decomposition of the incident plane-wave into a series of multimode spherical waves in the region of the sphere, as shown in Fig. S5, suitably modified to accommodate multipoles of high orbital angular number up to $l = 30$, as needed to obtain convergence beyond the scale of the plots. The individual spherical-wave modes are scattered by the sphere according to Mie theory, which we adapt to include the effect of the molecular coating on the Mie coefficients. The effect of the planar substrate is included via its Fresnel reflection and transmission coefficients. The scattered field is then evaluated in the far-field (both on the air and glass sides), from which the scattering cross section is calculated, taking into account the respective numerical apertures used in experiment for air and glass collection (0.8 and 0.6, respectively, see Fig. S1).

Molecular layers are modelled with a dielectric permittivity characterized by a Lorentzian term as

$$\epsilon_{\mathrm{S2275}}(E) = \epsilon_\infty + \frac{f E_{\mathrm{res}}^2}{E_{\mathrm{res}}^2 - E^2 - iE\gamma_{\mathrm{res}}}, \tag{1}$$

where $\epsilon_\infty = 1.9$ is the background permittivity, f is the reduced oscillator strength, $E_{\mathrm{res}}$is the photon energy for maximum molecular absorption, and γ_{res} is the linewidth of such absorption peak (see parameter values in the main text). We vary f to fit the absorption spectra observed for four layers of S2275 dye molecules on a glass substrate (see Fig. 1(b) in the main text) and find $f = 0.5$ to be a good value for this parameter. The nominal thickness of each PDAC/S2275 dye molecules is set to 4 nm.

S3.1 Peak broadening

To phenomenologically account for morphological imperfections, losses, and limitations in the resolution of the detector in the experiments –which results in spectral broadening–, we convolute the calculated scattering

efficiency at each wavelength with a Gaussian function with varying FWHM. In Figures S6 and S7, we show the result of the convolution to the data presented in Fig. 4 of the main text using a Gaussian with 10 nm and 2 nm FWHM, respectively (corresponding to approximately 20−40 and 4−8 meV, respectively, from the highest to the lowest wavelength values). Comparing Figures S6 and S7 with Figure 4 in the main text, we see how the artificially-introduced broadening removes the sharp resonance peaks and generally improves the agreement between the simulations and the experimental data in Figs. 2 and 3 in the main text.

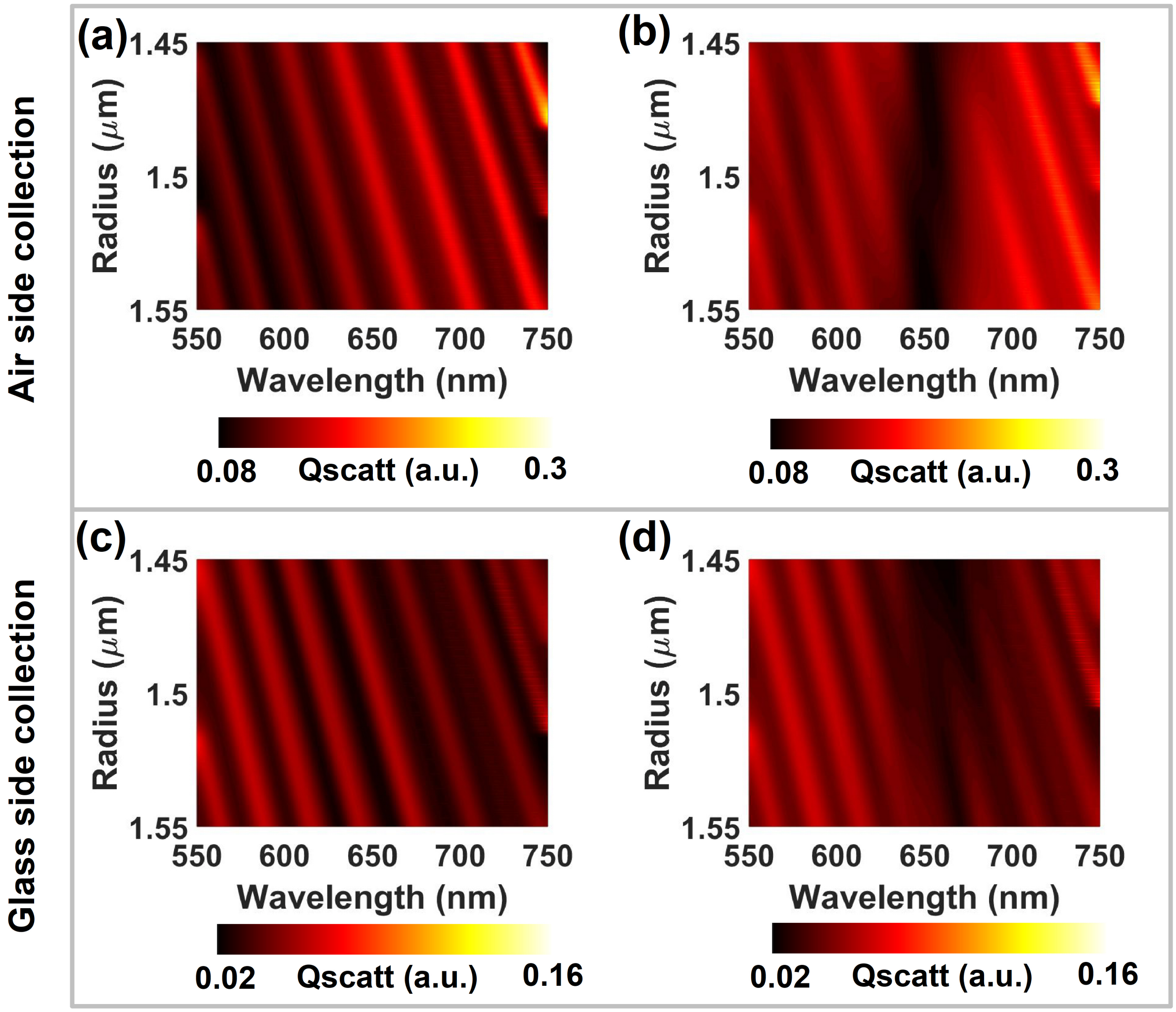

Figure S6: Numerically calculated dispersion of the air-side scattering efficiency of (a) a *bare* microsphere and (b) a microsphere coated with four layers of S2275 dye, both of them placed on a planar glass substrate. The results are convoluted with a Gaussian function of 10 nm FWHM (corresponding to approximately 20−40 meV, depending on the wavelength) to effectively account for experimental broadening effects. (c,d) Analogous to (a) and (b), respectively, but for glass-side scattering. In all cases, the angle of illumination is kept at 20° with respect to the substrate plane.

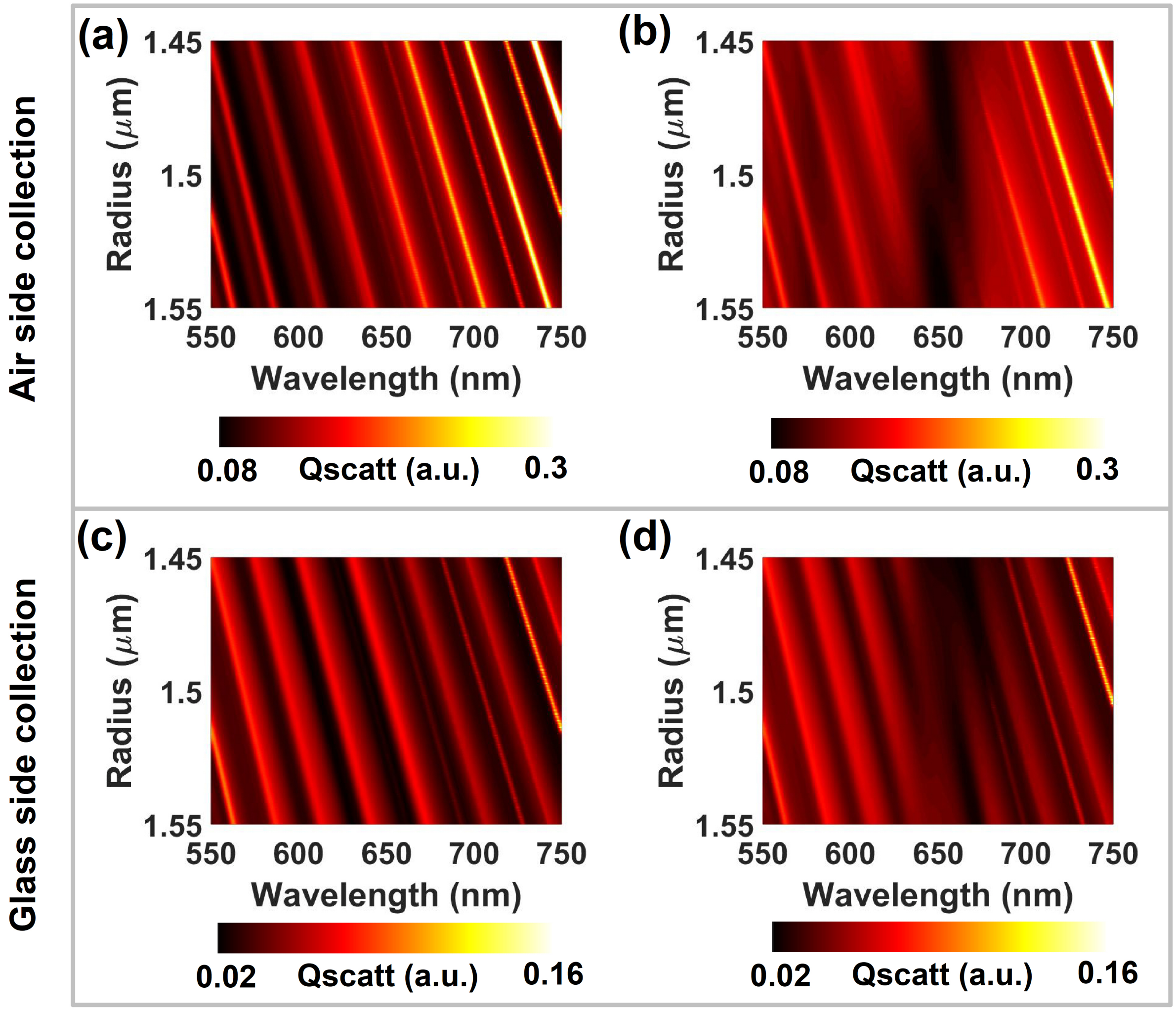

Figure S7: Numerically calculated dispersion of the air-side scattering efficiency of (a) a *bare* microsphere and (b) a microsphere coated with four layers of S2275 dye, both of them placed on a planar glass substrate. The results are convoluted with a Gaussian function of 2 nm FWHM (corresponding to approximately 4−8 meV, depending on the wavelength) to effectively account for experimental broadening effects. (c,d) Analogous to (a) and (b), respectively, but for glass-side scattering. In all cases, the angle of illumination is kept at 20° with respect to the substrate plane.

S3.2 Electric-field profiles

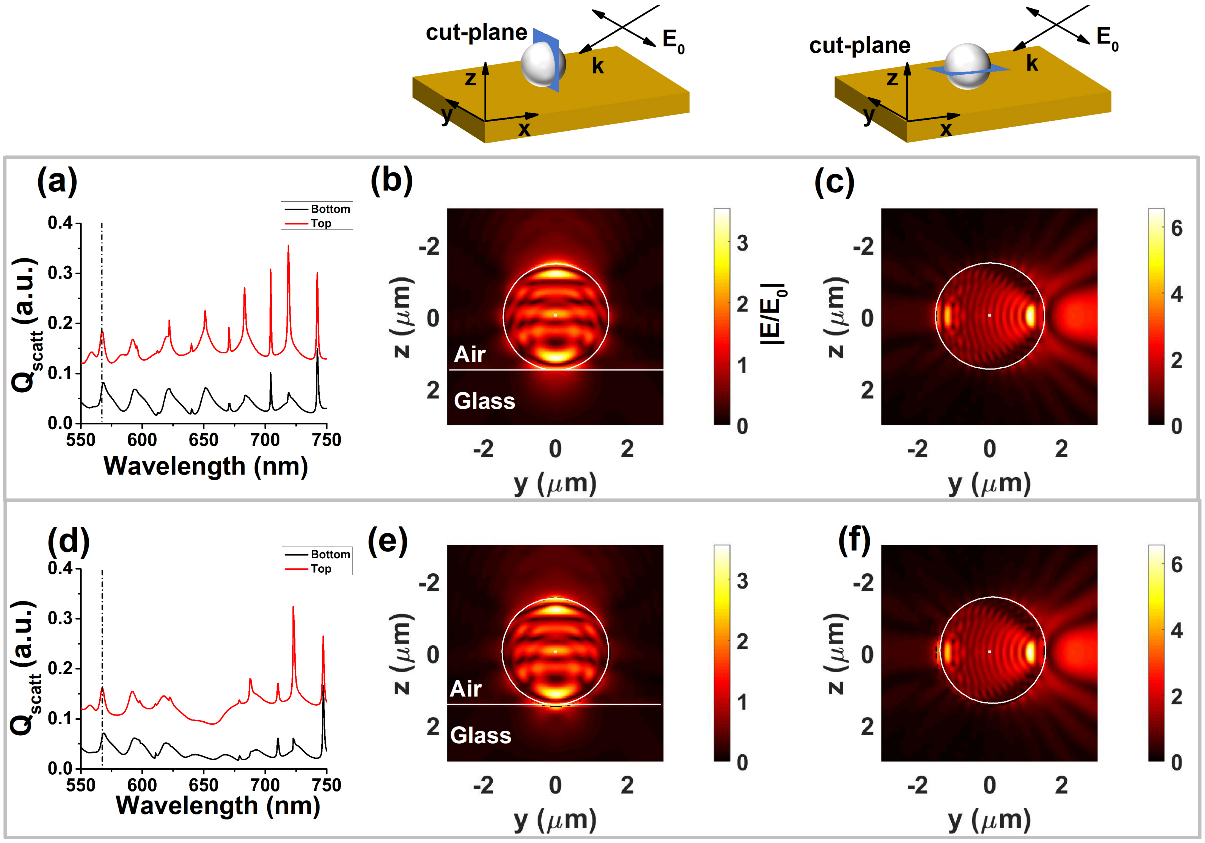

Figure S8: (a) Numerically calculated scattering spectra of a bare microsphere of 1.5 μm radius on a glass substrate, respectively for air-side (red-solid curve) and glass-side (black-solid curve) collection, under illumination by a plane-wave propagating as indicated in the schemes above the plots. The black-dashed line represents the excitation wavelength used in the calculation of the electric field (567 nm). (b,c) Numerically calculated scattered electric-field enhancement profiles for a bare microsphere of 1.5 μm radius placed on a glass substrate along either (b) the $x = 0$ plane or (c) the $z = 0$ plane (see cross-sectional cuts on the schemes above the corresponding plots). (d,e,f) Analogous to panels (a,b,c), respectively, but for a microsphere of radius 1.5 μm coated with four layers of S2275 dye.

S3.3 Molecule-cavity coupling in unsupported microspheres

Figures S9 shows the numerically calculated dispersion of an unsupported bare polystyrene microsphere in air, and a microsphere coated with four layers of S2275 molecules, respectively. There is a clear perturbation in the scattering modes of the microsphere when the dye molecules are coating the surface (cf. Figs. S9(a) and (b)). However, because of the smudging of the mode structure, we cannot clearly resolve the signatures of molecule-sphere strong coupling. When the microsphere is placed on a glass substrate, the symmetry of the structure is broken, and we can see a different outcoupling of the modes when observing from either the air side or the glass side, revaling a clear molecule-cavity coupling in the former. Further, to clearly resolve the mode structure for airborne spheres, we artificially reduce the absorption linewidth of the molecular coating down to 10 meV (see Fig. S9(c)), which produces clear signatures of strong coupling with molecule-cavity coupling strength of $2g$=90 meV.

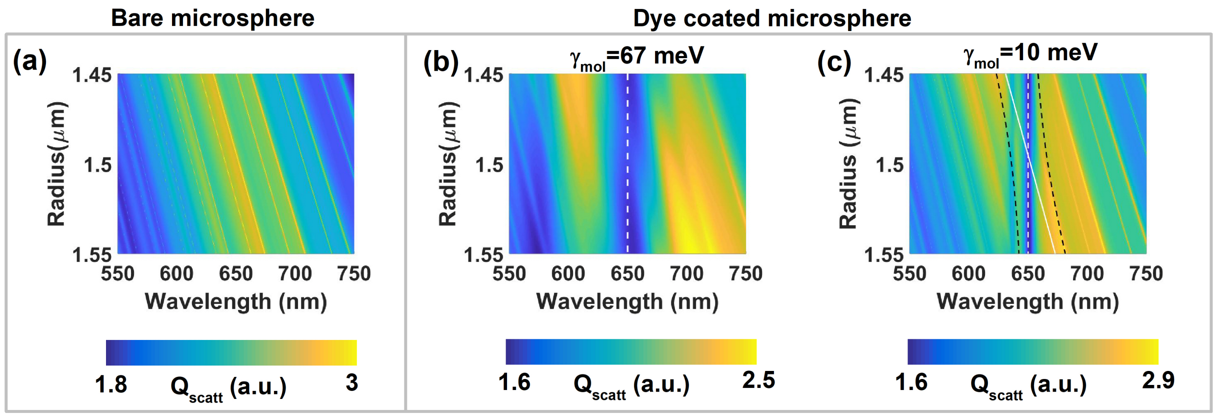

Figure S9: (a) Numerically calculated scattering efficiency of an unsupported bare polystyrene microsphere in air. (b) and (c) Same as (a), but for a microsphere coated with four layers of dye molecules that feature an absorption linewidth of 67 meV and 10 meV, respectively. In panel (c), a coupled-oscillator model fit is superimposed as dashed-black lines to quantify the molecule-cavity coupling strength. The solid-white line represents the bare cavity modes of the microsphere and the dashed-white line indicates the molecular absorption wavelength.

References

[1] Vasista, A. B. & Barnes, W. L. Strong Coupling of Multimolecular Species to Soft Microcavities. *J. Phys. Chem. Lett.* **13**, 1019–1024 (2022).

[2] Vasista, A. B. & Barnes, W. L. Molecular Monolayer Strong Coupling in Dielectric Soft Microcavities. *Nano Lett.* **20**, 1766–1773 (2020).

[3] Kühler, P. *et al.* Imprinting the Optical Near Field of Microstructures with Nanometer Resolution. *Small* **5**, 1825–1829 (2009).

[4] García de Abajo, F. J., Gómez-Santos, G., Blanco, L. A., Borisov, A. G. & Shabanov, S. V. Tunneling Mechanism of Light Transmission through Metallic Films. *Phys. Rev. Lett.* **95**, 067403 (2005).
